# Correction: Associations between physical activity, physical fitness, and body composition in adults living in Germany: A cross-sectional study

**DOI:** 10.1371/journal.pone.0341576

**Published:** 2026-01-21

**Authors:** Raphael Schilling, Steffen C. E. Schmidt, Janis Fiedler, Alexander Woll

In the Physical fitness subsection of the Materials and methods, the equation shows an incorrect formula for calculating the Z-scores. Please view the complete, correct equation here:


$$\begin{array}{c} \text{Z}\;=\;\text{100}\;+\;\frac{\text{raw}\;\text{value}\;-\;\overset{―}{\text{x}}}{\overset{―}{\text{s}}}\;•\;\text{10} \end{array}$$

$\overset{―}{\text{x}}$ = mean value of the 33-36-year-old men at the first time of measurement in 1992,

$\overset{―}{\text{s}}$ = standard deviation of the 33-36-year-old men at the first time of measurement in 1992
