# Supplementary material for: Associations between physical activity, physical fitness, and body composition in adults living in Germany: A cross-sectional study
Source: PLoS One. 2023 Oct 26;18(10):e0293555. doi: 10.1371/journal.pone.0293555 (PMC10602354; doi:10.1371/journal.pone.0293555)
Supplement: S1 Checklist — (DOCX) [file pone.0293555.s001.docx]

STROBE Statement—checklist of items that should be included in reports of observational studies

|  | Item No. | Recommendation | Page  No. | Relevant text from manuscript |
| --- | --- | --- | --- | --- |
| **Title and abstract** | 1 | (*a*) Indicate the study’s design with a commonly used term in the title or the abstract | 1 | Associations between physical activity, physical  fitness, and body composition in adults living in  Germany: A cross-sectional study |
|  |  | (*b*) Provide in the abstract an informative and balanced summary of what was done and what was found | 1-2 | Methods: Data from 329 men and women aged 35 to 86 years were analyzed. PA was measured by questionnaire and classified into sport activity and habitual activity. PF was measured through physical performance tests and BC by bioelectrical impedance analysis. Fat mass index (FMI) and fat-free mass index (FFMI) were calculated to represent height-adjusted BC. Associations between PA, PF, and BC were analyzed using linear regression models.  Results: For both sexes, strength was positively associated with FFMI (♂: ß = 0.313; ♀: ß = 0.213) and phase angle (♂: ß = 0.357; ♀: ß = 0.409). For FMI, a significant, negative association with strength was found only in women (ß = -0.189). Cardiorespiratory fitness showed a negative association with FMI (ß = -0.312) and FFMI (ß = -0.201) for men, while in women a positive association was found for FFMI (ß = 0.186). For coordination, a significant association with FMI was observed only in women (ß = -0.190). Regarding PA only one significant relationship between sport activity and FMI among women (ß = -0.170) was found. |
| Introduction | | | |  |
| Background/rationale | 2 | Explain the scientific background and rationale for the investigation being reported | 2-3 | Nowadays, external influences like digital and social media as well as new technologies change our daily routines, PA and lifestyle faster than ever and it is important to analyze which behaviors are beneficial for successful aging. In addition, the world's older population is growing dramatically [33]. With an aging population and the associated changes in BC, it is important to determine how PA, PF, and BC are associated with aging and themselves. Current cross-sectional studies already indicate that both PA [34–38] and PF [39, 40] are associated with BC in adulthood. Although, except for one study [36], BC is recorded with technical procedures such as bioelectrical impedance analysis [37, 38] or dual-energy X-ray absorptiometry [34, 35, 39, 40], only a few studies use fat mass index (FMI, as of fat mass (kg)/height2 (m)), and fat-free mass index (FFMI, as of fat-free mass (kg)/height2 (m)) to describe BC. Recent literature suggests that there is a rationale for using FFMI and FMI instead of body fat percentage for tracking healthy BC, especially when comparing data gathered through different technical procedures or identifying clinical phenotypes like Sarcopenia [41]. Additionally, only a few studies look at the interdependency of all three constructs: PA, PF, and BC. In this context, the WHO states specifically that more research is needed on the relationship between PA and health outcomes [26]. |
| Objectives | 3 | State specific objectives, including any prespecified hypotheses | 3 | In this study, we used a cross-sectional design to describe the age-related development of BC among adults and to examine the sex-specific associations between PA, PF, and BC. |
| Methods | | | |  |
| Study design | 4 | Present key elements of study design early in the paper | 4 | All data originates from a community-based, longitudinal German study [42] with currently six measurements in 1992, 1997, 2002, 2010, 2015, and 2021. The present cross-sectional analysis refers to the 2021 measurement point. |
| Setting | 5 | Describe the setting, locations, and relevant dates, including periods of recruitment, exposure, follow-up, and data collection | 4 | For the 2021 measurement, n=666 participants that participated in earlier waves were invited to join the study again and n=300 did so (response: 45,1%). In addition, a new cross-sectional cohort of 300 35-year-olds (response: 14,7%) and 300 55-year-olds (response: 23,3%) were randomly selected from the residents’ registration offices in Bad Schönborn, Germany. In total, 430 adults participated in 2021 and the age range was 35 to 86 years. Participation was voluntary and participants provided their written consent to participate in the study. The applied protocols were approved by a scientific advisory council, the Schettler Clinic, Bad Schönborn, Germany as well as the ethics committee of the Karlsruhe Institute of Technology. We strictly followed ethical guidelines from the German Psychological Society. The 2021 measurement took place during a low-incidence time frame of the COVID-19 pandemic in Germany from June 21st, 2021 to July 29th, 2021. For the present analyses, 88 subjects were excluded due to missing data in the variables of interest, and 13 subjects were excluded due to extreme values in the study variables. Finally, a sample of 329 participants was examined in this study. |
| Participants | 6 | (*a*) *Cohort study*—Give the eligibility criteria, and the sources and methods of selection of participants. Describe methods of follow-up  *Case-control study*—Give the eligibility criteria, and the sources and methods of case ascertainment and control selection. Give the rationale for the choice of cases and controls  *Cross-sectional study*—Give the eligibility criteria, and the sources and methods of selection of participants | 4 | For the 2021 measurement, n=666 participants that participated in earlier waves were invited to join the study again and n=300 did so (response: 45,1%). In addition, a new cross-sectional cohort of 300 35-year-olds (response: 14,7%) and 300 55-year-olds (response: 23,3%) were randomly selected from the residents’ registration offices in Bad Schönborn, Germany. In total, 430 adults participated in 2021 and the age range was 35 to 86 years. Participation was voluntary and participants provided their written consent to participate in the study. The applied protocols were approved by a scientific advisory council, the Schettler Clinic, Bad Schönborn, Germany as well as the ethics committee of the Karlsruhe Institute of Technology. We strictly followed ethical guidelines from the German Psychological Society. The 2021 measurement took place during a low-incidence time frame of the COVID-19 pandemic in Germany from June 21st, 2021 to July 29th, 2021. For the present analyses, 88 subjects were excluded due to missing data in the variables of interest, and 13 subjects were excluded due to extreme values in the study variables. Finally, a sample of 329 participants was examined in this study. |
|  |  | (*b*) *Cohort study*—For matched studies, give matching criteria and number of exposed and unexposed  *Case-control study*—For matched studies, give matching criteria and the number of controls per case | - | - |
| Variables | 7 | Clearly define all outcomes, exposures, predictors, potential confounders, and effect modifiers. Give diagnostic criteria, if applicable | 4-7 | The questionnaire differentiates between sport activity and habitual activity. Sport activity includes all physical-sporting activities that can be assigned to sports or exercising. In addition to the general question "Do you engage in sports or gymnastic exercises?", the types of sports were asked, as well as the minutes per week and the number of weeks per year in which the activity is pursued. The max-imum weekly duration was set to 900 minutes per sport and values above 900 minutes for one sport activity were trimmed to 900. The total minutes of sport activity is the sum of all minutes in the speci-fied sports, multiplied by the concordant weeks per year, divided by 52. For sports that do not cause a noticeable increase in energy expenditure by large skeletal muscle (e.g. chess, esports), the duration was set to zero. To estimate habitual activity, the minutes of weekly walking and cycling for transportation as well as other exhausting habitual activities such as gardening were collected.  Daily walking was determined by asking about the distance walked on a typical weekday. Possible answers were: "I almost never walk ≙ 0km", "Less than 1km/day (only in the house) ≙ 0.5km", "1-2 km/day (in the house and shorter walking distances) ≙ 1.5km", "3-5 km/day (longer walking distances away from home) ≙ 4km", "6-9 km/day ≙ 7km", "10km/day and more ≙ 12km". The resulting average distances were multiplied by a factor of 5.25*60 to obtain an average duration in minutes. Daily cycling was raised through a question of whether the participant uses a bicycle for transportation and if the answer was yes, a second item asked about how many minutes they usually cycle daily. Experience showed that for most participants, the minutes of daily cycling can be remembered or added up much easier than the duration of daily walking. Exhausting leisure activities were asked in the form of two items, "Do you perform other physically demanding leisure activities (e.g., gardening)?" and "Overall, how much time do you spend doing this (other than exercising) in minutes per week?". Here, we did not ask about the daily minutes, but the weekly minutes, since these activities often take place far more irregularly compared to the distances traveled. Finally, converting strenuous leisure activities into minutes per day and adding the minutes spent walking and cycling resulted in an index of habitual activity for all participants  Twelve motor performance tests were used to assess PF in 2021. All tests were performed during a single session chronologically after the assessment of BC and were supervised, following a standardized test protocol with acceptable reliability [45]. Cardiorespiratory fitness (CRF) was measured by maximal oxygen uptake (VO2max) estimated from time for completion and heart rate during a 2-km walking test [46]. Strength was assessed by a handgrip (digital hand dynamometer, 198 lbs, GRIPX, China) and a jump-and-reach test. Coordination was measured by standing on one leg with closed eyes, whilst moving the second leg in circles, and three test items with balls: Throw against a wall and catch (exteroceptive, ballistic), throw with rotation and catch (exteroceptive, ballistic, pressure of time), as well as a task where you hold a ball between your legs with one hand in front of the thigh and the other hand behind the other thigh, then releasing and catching the ball with changing the grip five times (interoceptive, tactile-ballistic) [45]. Every task was judged according to standardized rules by the instructors, differentiating among ‟2 - task solved easily‟, ‟1 = task solved with problems‟ and ‟0 = task not solved‟. Finally, a coordination index (0-8) of the added-up points was calculated for each participant. Flexibility was measured by a sit-and-reach test and a trunk side bending test, as well as an index for muscle shortening derived from six three-scaled (2 points = no restrictions, 1 point = slight restrictions, 0 points = major restrictions) items for shoulder neck mobility (left/right), hamstring (left/right), and rectus femoris (left/right) muscle extensibility. The index for muscle shortening ranged from 0 to 12 and was obtained by adding up the achieved points in shoulder neck mobility (left/right), hamstring (left/right), and rectus femoris (left/right).  For handgrip, jump-and-reach, sit-and-reach test, and coordination items, the best of two trials was used in the analyses, all other items were performed one time. The test battery was developed in cooperation with the UKK Institute in Tampere, Finland [45]. Because items with different units were analyzed, a Z-score transformation was used for VO2max, handgrip-strength, jump-and-reach-heights, achieved points in the coordination test, and achieved points in the muscle shortening test battery. These items were standardized according to Woll et al. [43] based on male participants aged between 33 and 36 years in 1992. Thus, the performance of 35-year-old males in 1992 is Z=100 in all test items of motor performance. The Z-value transformation results as follows:  "Z = 100 + " ("raw value - " "x" ̅)/"s" ̅ " • 10"  x̅ = mean value of the 33-36-year-old men at the first time of measurement in 1992  s̅ = standard deviation of the 33-36-year-old men at the first time of measurement in 1992  Finally, indices for strength (mean Z-value from handgrip and jump-and-reach), CRF (Z-value of the VO2max), coordination (Z-value of the coordination index), and flexibility (Z-value of the flexibility index) were calculated and used for the latter analyses.  From bioelectrical impedance analysis, we used PhA and calculated FMI and FFMI. FMI was calculated as fat mass (kg)/height2 (m) and FFMI as fat-free mass (kg)/height2 (m). Fat mass and fat-free mass were derived from the seca formulas with age- and sex-related in-house reference data for resistance and reactance, which are not publicly accessible. |
| Data sources/ measurement | 8* | For each variable of interest, give sources of data and details of methods of assessment (measurement). Describe comparability of assessment methods if there is more than one group | 4-7 | PA was assessed via digital questionnaires that were filled in on computers in the survey center. The questionnaire was proofed in relation to reliability (test-retest after two weeks: r>.90 and Cronbach’s α=.94), factorial validity, and measurement invariance [44]. The questionnaire differentiates between sport activity and habitual activity.  Twelve motor performance tests were used to assess PF in 2021. All tests were performed during a single session chronologically after the assessment of BC and were supervised, following a standardized test protocol with acceptable reliability [45]. Cardiorespiratory fitness (CRF) was measured by maximal oxygen uptake (VO2max) estimated from time for completion and heart rate during a 2-km walking test [46]. Strength was assessed by a handgrip (digital hand dynamometer, 198 lbs, GRIPX, China) and a jump-and-reach test. Coordination was measured by standing on one leg with closed eyes, whilst moving the second leg in circles, and three test items with balls: Throw against a wall and catch (exteroceptive, ballistic), throw with rotation and catch (exteroceptive, ballistic, pressure of time), as well as a task where you hold a ball between your legs with one hand in front of the thigh and the other hand behind the other thigh, then releasing and catching the ball with changing the grip five times (interoceptive, tactile-ballistic) [45]. Every task was judged according to standardized rules by the instructors, differentiating among ‟2 - task solved easily‟, ‟1 = task solved with problems‟ and ‟0 = task not solved‟. Finally, a coordination index (0-8) of the added-up points was calculated for each participant. Flexibility was measured by a sit-and-reach test and a trunk side bending test, as well as an index for muscle shortening derived from six three-scaled (2 points = no restrictions, 1 point = slight restrictions, 0 points = major restrictions) items for shoulder neck mobility (left/right), hamstring (left/right), and rectus femoris (left/right) muscle extensibility. The index for muscle shortening ranged from 0 to 12 and was obtained by adding up the achieved points in shoulder neck mobility (left/right), hamstring (left/right), and rectus femoris (left/right).  For handgrip, jump-and-reach, sit-and-reach test, and coordination items, the best of two trials was used in the analyses, all other items were performed one time. The test battery was developed in cooperation with the UKK Institute in Tampere, Finland [45]. Because items with different units were analyzed, a Z-score transformation was used for VO2max, handgrip-strength, jump-and-reach-heights, achieved points in the coordination test, and achieved points in the muscle shortening test battery. These items were standardized according to Woll et al. [43] based on male participants aged between 33 and 36 years in 1992. Thus, the performance of 35-year-old males in 1992 is Z=100 in all test items of motor performance. The Z-value transformation results as follows:  "Z = 100 + " ("raw value - " "x" ̅)/"s" ̅ " • 10"  x̅ = mean value of the 33-36-year-old men at the first time of measurement in 1992  s̅ = standard deviation of the 33-36-year-old men at the first time of measurement in 1992  Finally, indices for strength (mean Z-value from handgrip and jump-and-reach), CRF (Z-value of the VO2max), coordination (Z-value of the coordination index), and flexibility (Z-value of the flexibility index) were calculated and used for the latter analyses  We measured body height with a fixed stadiometer (seca 213, seca gmbh, Germany) and waist circumference by measuring tape through trained personnel. The seca medical body composition analyzer (seca mBCA 515) was used to measure body weight and BC according to the international ES-PEN standards [47]. According to ESPEN standards, the absence of drugs but not fasting was a pre-requisite and participants were invited to join the study with normal nutritional status |
| Bias | 9 | Describe any efforts to address potential sources of bias | - | - |
| Study size | 10 | Explain how the study size was arrived at | 4 | The 2021 measurement took place during a low-incidence time frame of the COVID-19 pandemic in Germany from June 21st, 2021 to July 29th, 2021. For the present analyses, 88 subjects were excluded due to missing data in the variables of interest, and 13 subjects were excluded due to extreme values in the study variables. Finally, a sample of 329 participants was examined in this study. |

Continued on next page

| Quantitative variables | 11 | Explain how quantitative variables were handled in the analyses. If applicable, describe which groupings were chosen and why | 7-8 | The IBM© SPSS© Statistics package (Version 28.0) was used for the analysis. To present basic descriptive statistics, mean values and standard deviations were calculated for all study variables. As previously described, subjects with extreme values were excluded. In the present work, extreme values for men and women, respectively, were defined as data points that were more than 3 standard deviations away from the upper or lower limit of the interquartile range. Pearson product-moment correlation coefficients (r) were performed to examine the relationship between PA, PF, BC, and age. To visualize the relationships between FMI, FFMI, and PA with age, scatter plots were created for men and women. Regression models were performed to examine the cross-sectional associations of age, PA, and PF with FMI, FFMI, and PhA. The final models consisted of age (Model 0), sport activity, habitual activity (Model 1), strength, coordination, flexibility, and CRF (Model 2) with FMI, FFMI, and PhA as the dependent variables. Age was also included as an additional predictor in Model 1 (Model 1.2) and Model 2 (Model 2.1). Lastly, in Model 3, all predictors were considered in one model. The analyzes were carried out separately for each sex. Since we do not assume a linear change of BC during aging, polynomial regression models were calculated for all parameters of BC and age. From those analyses, only PhA among women showed a significant negative quadratic decline. We show this polynomial curve progression in Fig 1c and added the quadratic age coefficient in Table 3. Age was grand mean centered at the average age of all participants. In the end, six models each were calculated for men and women for the dependent variables FMI, FFMI, and PhA. Statistical signifi-cance was set a priori at the level of p ≤ 0.05. |
| --- | --- | --- | --- | --- |
| Statistical methods | 12 | (*a*) Describe all statistical methods, including those used to control for confounding | 7-8 | The IBM© SPSS© Statistics package (Version 28.0) was used for the analysis. To present basic descriptive statistics, mean values and standard deviations were calculated for all study variables. As previously described, subjects with extreme values were excluded. In the present work, extreme values for men and women, respectively, were defined as data points that were more than 3 standard deviations away from the upper or lower limit of the interquartile range. Pearson product-moment correlation coefficients (r) were performed to examine the relationship between PA, PF, BC, and age. To visualize the relationships between FMI, FFMI, and PA with age, scatter plots were created for men and women. Regression models were performed to examine the cross-sectional associations of age, PA, and PF with FMI, FFMI, and PhA. The final models consisted of age (Model 0), sport activity, habitual activity (Model 1), strength, coordination, flexibility, and CRF (Model 2) with FMI, FFMI, and PhA as the dependent variables. Age was also included as an additional predictor in Model 1 (Model 1.2) and Model 2 (Model 2.1). Lastly, in Model 3, all predictors were considered in one model. The analyzes were carried out separately for each sex. Since we do not assume a linear change of BC during aging, polynomial regression models were calculated for all parameters of BC and age. From those analyses, only PhA among women showed a significant negative quadratic decline. We show this polynomial curve progression in Fig 1c and added the quadratic age coefficient in Table 3. Age was grand mean centered at the average age of all participants. In the end, six models each were calculated for men and women for the dependent variables FMI, FFMI, and PhA. Statistical signifi-cance was set a priori at the level of p ≤ 0.05. |
|  |  | (*b*) Describe any methods used to examine subgroups and interactions | - | - |
|  |  | (*c*) Explain how missing data were addressed | 4 & 7 | For the present analyses, 88 subjects were excluded due to missing data in the variables of interest, and 13 subjects were excluded due to extreme values in the study variables.  In the present work, extreme values for men and women, respectively, were defined as data points that were more than 3 standard deviations away from the upper or lower limit of the interquartile range |
|  |  | (*d*) *Cohort study*—If applicable, explain how loss to follow-up was addressed  *Case-control study*—If applicable, explain how matching of cases and controls was addressed  *Cross-sectional study*—If applicable, describe analytical methods taking account of sampling strategy | - | - |
|  |  | (*e*) Describe any sensitivity analyses | - | - |
| Results | | | | |
| Participants | 13* | (a) Report numbers of individuals at each stage of study—eg numbers potentially eligible, examined for eligibility, confirmed eligible, included in the study, completing follow-up, and analysed | 8 | Table 1 |
|  |  | (b) Give reasons for non-participation at each stage | - | - |
|  |  | (c) Consider use of a flow diagram | - | - |
| Descriptive data | 14* | (a) Give characteristics of study participants (eg demographic, clinical, social) and information on exposures and potential confounders | 8 | The main characteristics of the sample are presented in Table 1. Despite the higher body weight in men, women had a higher FM. FFMI and PhA were in a higher range for men while FMI was higher in women. Men showed higher values for habitual activity, while women engaged more often in sports activities. Concerning PF, there were higher values in strength, coordination, and CRF in men, while women performed better in flexibility |
|  |  | (b) Indicate number of participants with missing data for each variable of interest | - | - |
|  |  | (c) *Cohort study*—Summarise follow-up time (eg, average and total amount) | - | - |
| Outcome data | 15* | *Cohort study*—Report numbers of outcome events or summary measures over time | - | - |
|  |  | *Case-control study—*Report numbers in each exposure category, or summary measures of exposure | - | - |
|  |  | *Cross-sectional study—*Report numbers of outcome events or summary measures | 8-12 | Table 1; Table2&3; Fig1 |
| Main results | 16 | (*a*) Give unadjusted estimates and, if applicable, confounder-adjusted estimates and their precision (eg, 95% confidence interval). Make clear which confounders were adjusted for and why they were included | 9-12 |  |
|  |  | (*b*) Report category boundaries when continuous variables were categorized | - | - |
|  |  | (*c*) If relevant, consider translating estimates of relative risk into absolute risk for a meaningful time period | - | - |

Continued on next page

| Other analyses | 17 | Report other analyses done—eg analyses of subgroups and interactions, and sensitivity analyses | - | - |
| --- | --- | --- | --- | --- |
| Discussion | | | | |
| Key results | 18 | Summarise key results with reference to study objectives | 13 | The goal of this study was to describe BC during adulthood using a cross-sectional sample from a German community and to investigate the associations between PA, PF, and BC under consideration of age. Our results confirm a relationship between age and FMI, FFMI, as well as PhA. FMI increased with age for both sexes, whereas PhA decreased. FFMI decreased significantly among women, but not among men and the decrease of PhA among women showed a significant quadratic term, indicating an even increasing decline with increasing age. Furthermore, the results show that there was an as-sociation between different parameters of PF and BC for both sexes. In particular, strength and CRF were associated with BC. Regarding PA, only one significant relationship between sport activity and FMI was found among women. No associations with BC could be detected for habitual activity |
| Limitations | 19 | Discuss limitations of the study, taking into account sources of potential bias or imprecision. Discuss both direction and magnitude of any potential bias | 18 | There are limitations to this study. First, due to the chosen cross-sectional design no causal state-ments can be made. The results thus provide information at a single point in time. Second, nutrition was not recorded. Nutrition is considered one of the main influencing factors for BC [96] and may influence the relationship between PA, PF, and BC. Therefore, the complex, multidirectional interrelation between BC, PF, and PA and their correlates endorses that in addition to an active lifestyle, other risk factors that lead to poor BC, poor PF, or physical inactivity like hyper-caloric nutrition, malnutri-tion, or specific diseases, should be considered in further studies. Third, PA was assessed using self-reports, which are associated with several problems such as recall bias [97] and over- or underestimation of the activity level [98]. For this reason, device-based measurements such as accelerometry are often used and should be considered to replicate our findings regarding a relatively low influence of PA on BC |
| Interpretation | 20 | Give a cautious overall interpretation of results considering objectives, limitations, multiplicity of analyses, results from similar studies, and other relevant evidence | 18-19 | Our results indicate that among individuals in middle and later adulthood, PF is stronger related to BC than self-reported PA. Especially strength and to a lower extent CRF are associated with BC. This supports WHO's activity recommendations that adults should engage in aerobic and resistance training to maintain an improvement in strength and CRF and to counteract age-related declines, thus having a positive effect on BC besides nutrition alone. Resistance training, in particular, should be given a high priority with increasing age, as the clearest interrelations between PF and BC for both sexes were found for strength. Especially concerning Dynapenia, the age-related loss of strength plays a decisive role and should thus be increasingly considered when establishing PA recommendations. Here, PhA could serve as an indicator for the detection of the condition. We also assume that PA might have a more noticeable effect on BC and PF if the intensity is emphasized in addition to the overall volume of PA. Accordingly, it would be important to plan and structure the training sessions individually to achieve sustainable progression. However, further investigations must be carried out to confirm this in practice. Future research should examine the influences of PA and PF on BC using longitudinal analyses to draw causal conclusions and accelerometry should be used to replicate our results. |
| Generalisability | 21 | Discuss the generalisability (external validity) of the study results | 18  19 | Our results indicate that among individuals in middle and later adulthood, PF is stronger related to BC than self-reported PA…  Future research should examine the influences of PA and PF on BC using longitudinal analyses to draw causal conclusions and accelerometry should be used to replicate our results |
| Other information | |  | | |
| Funding | 22 | Give the source of funding and the role of the funders for the present study and, if applicable, for the original study on which the present article is based | 19 | The Authors acknowledge support by the KIT-Publication Fund of the Karlsruhe Institute of Technology. |

*Give information separately for cases and controls in case-control studies and, if applicable, for exposed and unexposed groups in cohort and cross-sectional studies.

**Note:** An Explanation and Elaboration article discusses each checklist item and gives methodological background and published examples of transparent reporting. The STROBE checklist is best used in conjunction with this article (freely available on the Web sites of PLoS Medicine at http://www.plosmedicine.org/, Annals of Internal Medicine at http://www.annals.org/, and Epidemiology at http://www.epidem.com/). Information on the STROBE Initiative is available at www.strobe-statement.org.
