# Supplementary figures and images for: Associations between physical activity, physical fitness, and body composition in adults living in Germany: A cross-sectional study
Source: PLoS One. 2023 Oct 26;18(10):e0293555. doi: 10.1371/journal.pone.0293555 (PMC10602354; doi:10.1371/journal.pone.0293555)

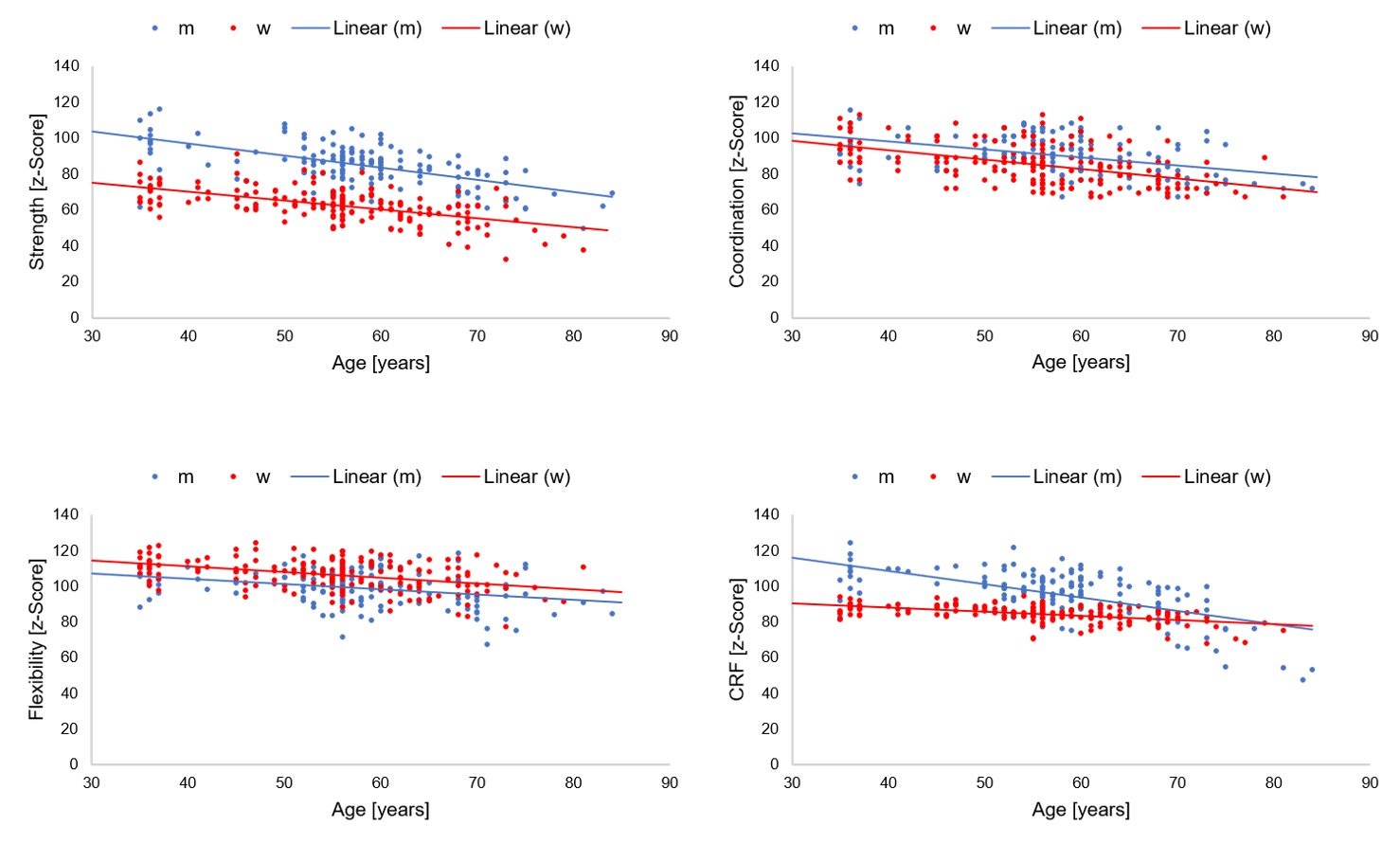

Supplement: S1 Fig — (TIF) [file pone.0293555.s002.tif]
