# Supplementary material for: Associations between physical activity, physical fitness, and body composition in adults living in Germany: A cross-sectional study
Source: PLoS One. 2023 Oct 26;18(10):e0293555. doi: 10.1371/journal.pone.0293555 (PMC10602354; doi:10.1371/journal.pone.0293555)
Supplement: S1 Table — (PDF) [file pone.0293555.s003.pdf]

S1Table: Correlation matrix for age, body composition, physical activity, and physical fitness

Men, n = 146<br>Women, n = 183		Age	FMI	FFMI	PhA	HA	SA	Strength	Coordination	Agility	CRF
Age	r	1	0.260**	-0.076	-0.558**	-0.012	0.146*	-0.601**	-0.422**	-0.312**	-0.593**
(years)	p.		<.001	.181	<.001	.445	.039	<.001	<.001	<.001	<.001
FMI	r	0.209**	1	0.493**	0.090	-0.017	-0.012	-0.250**	-0.227**	-0.161*	-0.368**
(kg/m^2)	p.	.002		<.001	.140	.421	.443	.001	.003	.026	<.001
FFMI	r	-0.238**	0.521**	1	0.356**	0.021	0.088	0.213**	-0.022	0.178*	-0.051
(kg/m^2)	p.	<.001	<.001		<.001	.401	.144	.005	.398	.016	.271
PhA	r	-0.419**	-0.071	0.495**	1	0.163*	0.085	0.480**	0.305**	0.282**	0.382**
(°)	p.	<.001	.171	<.001		.024	.154	<.001	<.001	<.001	<.001
HA	r	0.022	-0.100	-0.019	0.079	1	0.315**	0.050	-0.007	0.020	0.004
(min/week)	p.	.382	.088	.399	.143		<.001	.275	.467	.404	.483
SA	r	0.083	-0.183**	-0.064	0.033	0.205**	1	-0.053	-0.093	-0.062	0.090
(min/week)	p.	.131	.007	.194	.327	.003		.263	.133	.227	.140
Strength	r	-0.583**	-0.308**	0.223**	0.497**	0.045	-0.056	1	0.491**	0.439**	0.519**
(z-Score[a])	p.	<.001	<.001	.001	<.001	.271	.225		<.001	<.001	<.001
Coordination	r	-0.488**	-0.302**	0.023	0.292**	0.038	0.011	0.446**	1	0.402**	0.496**
(z-Score)	p.	<.001	<.001	.380	<.001	.305	.444	<.001		<.001	<.001
Agility	r	-0.393**	-0.270**	0.054	0.250**	0.008	-0.011	0.498**	0.382**	1	0.347**
(z-Score)	p.	<.001	<.001	.233	<.001	.457	.439	<.001	<.001		<.001
CRF	r	-0.519**	-0.181**	0.223**	0.373**	0.128*	0.072	0.550**	0.398**	0.408**	1
(z-Score)	p.	<.001	.007	.001	<.001	.042	.165	<.001	<.001	<.001	

Abbreviations: n, sample size; FMI, fat mass index; FFMI, fat-free mass index; PhA, phase angle; HA, habitual activity; SA, sport activity; CRF, cardiorespiratory fitness; kg, kilogram; m, meter; °, degree; min, minutes; r, correlation coefficient; sig., statistical significance.

*Correlation is significant at the 0.05 level (two-tailed).

**Correlation is significant at the 0.01 level (two-tailed).

[a]z-standardized variable.
